# Supplementary material for: Species and drug susceptibility profiles of staphylococci isolated from healthy children in Eastern Uganda
Source: PLoS One. 2020 Feb 13;15(2):e0229026. doi: 10.1371/journal.pone.0229026 (PMC7018018; doi:10.1371/journal.pone.0229026)
Supplement: S2 Table — (DOCX) [file pone.0229026.s002.docx]

**S2 Table: Details of drug resistance profiles of methicillin resistant staphylococci (n=122)**

| **Lab ID** | **Species** | **FOX** | **PEN** | **TET** | **SXT** | **ERY** | **CHL** | **GEN** | **CIP** | **RIF** | **CLI** | **MUP** | **TEI** | **LIN** | **VAN** | **DAP** | **SCC*mec*** | **MDR** |
| --- | --- | --- | --- | --- | --- | --- | --- | --- | --- | --- | --- | --- | --- | --- | --- | --- | --- | --- |
| 1. K-60 | *S. aureus* | **R** | **R** | **R** | **R** | **R** | S | S | **R** | S | S | S | S | S | S | S | I | **Yes** |
| 1. K-911 | *S. aureus* | **R** | **R** | **R** | S | **R** | S | **R** | **R** | S | S | S | S | S | S | S | I | **Yes** |
| 1. K-970 | *S. aureus* | **R** | **R** | **R** | **I** | S | S | S | **I** | S | S | S | S | S | S | S | I | No |
| 1. R-020 | *S. aureus* | **R** | **R** | S | **R** | S | S | **R** | S | S | S | S | S | S | S | S | I | **Yes** |
| 1. R-030 | *S. aureus* | **R** | **R** | **R** | **R** | **R** | S | **R** | **R** | S | S | S | S | S | S | S | I | **Yes** |
| 1. R-10 | *S. aureus* | **R** | **R** | S | **R** | **R** | S | S | **R** | S | S | S | S | S | S | S | I | **Yes** |
| 1. K914 | *S. aureus* | **R** | **R** | **R** | **R** | **R** | S | S | **R** | S | S | S | S | S | S | S | I | **Yes** |
| 1. K677 | *S. aureus* | **R** | **R** | **R** | **R** | S | S | S | **R** | S | S | S | S | S | S | S | I | **Yes** |
| 1. K642 | *S. aureus* | **R** | **R** | **R** | S | **R** | S | S | S | S | S | S | S | S | S | S | I | **Yes** |
| 1. K312-1 | *S. aureus* | **R** | **R** | **R** | S | **R** | S | S | **R** | S | S | S | S | S | S | S | I | **Yes** |
| 1. K460 | *S. aureus* | **R** | **R** | **R** | **R** | **R** | S | **R** | S | S | S | S | S | S | S | S | I | **Yes** |
| 1. K459 | *S. aureus* | **R** | **R** | **R** | S | **R** | S | S | S | S | S | S | S | S | S | S | I | **Yes** |
| 1. K439 | *S. aureus* | **R** | **R** | **R** | S | S | S | S | **R** | S | S | S | S | S | S | S | I | **Yes** |
| 1. K422 | *S. aureus* | **R** | **R** | **R** | **R** | **R** | S | S | S | S | S | S | S | S | S | S | I | **Yes** |
| 1. K282 | *S. aureus* | **R** | **R** | **R** | S | **R** | S | **R** | S | S | S | S | S | S | S | S | I | **Yes** |
| 1. K1057 | *S. aureus* | **R** | **R** | **R** | S | S | **R** | **R** | S | S | S | S | S | S | S | S | I | **Yes** |
| 1. K2401 | *S. aureus* | **R** | **R** | **R** | **R** | **R** | S | S | S | S | S | S | S | S | S | S | I | **Yes** |
| 1. K-4834 | *S. aureus* | **R** | **R** | **R** | S | S | **R** | **R** | S | S | S | S | S | S | S | S | I | **Yes** |
| 1. K1113 | *S. aureus* | **R** | **R** | S | S | S | S | **R** | S | S | S | S | S | S | S | S | I | No |
| 1. K1077 | *S. aureus* | **R** | **R** | S | **R** | S | S | **R** | S | S | S | S | S | S | S | S | I | **Yes** |
| 1. R-26A | *S. aureus* | **R** | **R** | S | **R** | **R** | S | S | **R** | S | S | S | S | S | S | S | I | **Yes** |
| 1. 306C | *S. aureus* | **R** | **R** | **R** | **R** | **R** | **I** | **R** | S | S | S | S | S | S | S | S | II | **Yes** |
| 1. K-2810 | *S. aureus* | **R** | **R** | **R** | **R** | **R** | S | S | **R** | S | S | S | S | S | S | S | II | **Yes** |
| 1. R-17 | *S. aureus* | **R** | **R** | **R** | **R** | S | S | **I** | S | S | S | S | S | S | S | S | II | **Yes** |
| 1. R33-1 | *S. aureus* | **R** | **R** | **R** | **R** | **R** | S | **R** | S | S | S | S | S | S | S | S | II | **Yes** |
| 1. K1131 | *S. aureus* | **R** | **R** | **R** | **R** | **R** | S | S | S | S | S | S | S | S | S | S | III | **Yes** |
| 1. 124 | *S. aureus* | **R** | **R** | S | S | S | S | S | **R** | S | S | S | S | S | S | S | IV | No |
| 1. 1322 | *S. aureus* | **R** | **R** | S | **R** | S | S | **R** | **I** | S | **I** | S | S | S | S | S | IV | No |
| 1. 1325 | *S. aureus* | **R** | **R** | S | **R** | S | S | **I** | S | S | S | S | S | S | S | S | IV | No |
| 1. 1326 | *S. aureus* | **R** | **R** | S | S | S | S | S | **R** | S | S | S | S | S | S | S | IV | No |
| 1. K-2240 | *S. aureus* | **R** | **R** | S | S | S | S | S | **R** | S | **I** | S | S | S | S | S | IV | No |
| 1. K-284 | *S. aureus* | **R** | **R** | S | **R** | **R** | S | **R** | S | S | S | S | S | S | S | S | IV | **Yes** |
| 1. R-31B | *S. aureus* | **R** | **R** | S | **R** | S | S | **R** | **R** | S | S | S | S | S | S | S | IV | **Yes** |
| 1. K-38 | *S. aureus* | **R** | **R** | S | **R** | S | S | **R** | **I** | S | S | S | S | S | S | S | IV | **Yes** |
| 1. K-39 | *S. aureus* | **R** | **R** | S | **R** | S | S | **R** | **R** | S | S | S | S | S | S | S | IV | **Yes** |
| 1. R-02 | *S. aureus* | **R** | **R** | S | **R** | S | S | S | **R** | S | S | S | S | S | S | S | IV | **Yes** |
| 1. R-040 | *S. aureus* | **R** | **R** | S | **R** | S | S | **R** | **R** | S | S | S | S | S | S | S | IV | **Yes** |
| 1. R-18 | *S. aureus* | **R** | **R** | S | S | S | S | S | **R** | S | S | S | S | S | S | S | IV | **Yes** |
| 1. R-20 | *S. aureus* | **R** | **R** | **I** | **R** | S | S | **R** | S | S | S | S | S | S | S | S | IV | **Yes** |
| 1. K526 | *S. aureus* | **R** | **R** | **R** | **R** | S | S | S | **R** | S | S | S | S | S | S | S | IV | **Yes** |
| 1. K544 | *S. aureus* | **R** | **R** | S | S | S | S | S | S | S | S | S | S | S | S | S | IV | No |
| 1. K1128 | *S. aureus* | **R** | **R** | S | S | S | S | S | S | S | S | S | S | S | S | S | IV | No |
| 1. R-22A | *S. aureus* | **R** | **R** | S | S | S | S | S | S | S | S | S | S | S | S | S | IV | No |
| 1. K-350C | *S. aureus* | **R** | **R** | S | S | S | S | S | **R** | S | S | S | S | S | S | S | V | No |
| 1. K-652 | *S. aureus* | **R** | **R** | S | S | S | **R** | **I** | S | S | S | S | S | S | S | S | V | No |
| 1. K1127 | *S. aureus* | **R** | **R** | S | S | S | S | S | S | S | S | S | S | S | S | S | V | No |
| 1. K662 | *S. aureus* | **R** | **R** | **R** | **R** | S | S | S | **R** | S | S | S | S | S | S | S | NT | **Yes** |
| 1. K525 | *S. aureus* | **R** | **R** | S | S | S | S | S | S | S | S | S | S | S | S | S | NT | No |
| 1. K299 | *S. aureus* | **R** | **R** | S | S | S | S | S | S | S | S | S | S | S | S | S | NT | No |
| 1. K261 | *S. aureus* | **R** | **R** | S | **R** | S | S | S | S | S | S | S | S | S | S | S | NT | No |
| 1. K1143 | *S. aureus* | **R** | **R** | S | **R** | S | S | S | S | S | S | S | S | S | S | S | NT | No |
| 1. K1048 | *S. aureus* | **R** | **R** | S | S | S | S | S | S | S | S | S | S | S | S | S | NT | No |
| 1. K1036 | *S. aureus* | **R** | **R** | S | **R** | S | S | S | S | S | S | S | S | S | S | S | NT | No |
| 1. K923-1 | *S. epidermidis* | **R** | **R** | **R** | S | **R** | S | **R** | S | S | S | S | S | S | S | S | I | **Yes** |
| 1. K685 | *S. epidermidis* | **R** | **R** | **R** | **R** | **R** | S | S | **R** | S | S | S | S | S | S | S | I | **Yes** |
| 1. K63-1 | *S. epidermidis* | **R** | **R** | **R** | **R** | **R** | S | S | **R** | S | S | S | S | S | S | S | I | **Yes** |
| 1. K1082 | *S. epidermidis* | **R** | **R** | **R** | **R** | **R** | S | **R** | S | S | S | S | S | S | S | S | I | **Yes** |
| 1. K269 | *S. epidermidis* | **R** | **R** | **R** | **R** | S | S | **R** | S | S | S | S | S | S | S | S | I | **Yes** |
| 1. K013-1 | *S. epidermidis* | **R** | **R** | **I** | **R** | **R** | S | S | S | S | **R** | S | S | S | S | S | I | **Yes** |
| 1. K940-2 | *S. epidermidis* | **R** | **R** | **R** | **R** | **R** | S | S | **R** | S | S | S | S | S | S | S | II | **Yes** |
| 1. K1106 | *S. epidermidis* | **R** | **R** | **R** | S | **R** | S | S | S | S | S | S | S | S | S | S | II | **Yes** |
| 1. K1059 | *S. epidermidis* | **R** | **R** | **R** | S | **R** | S | S | S | S | S | S | S | S | S | S | II | **Yes** |
| 1. K616 | *S. epidermidis* | **R** | **R** | **R** | S | S | S | **R** | S | S | S | S | S | S | S | S | III | **Yes** |
| 1. K614 | *S. epidermidis* | **R** | **R** | S | **R** | S | S | S | **R** | S | S | S | S | S | S | S | III | **Yes** |
| 1. K1103 | *S. epidermidis* | **R** | **R** | S | **R** | S | S | S | S | S | S | S | S | S | S | S | IV | No |
| 1. K931-3 | *S. epidermidis* | **R** | **R** | S | S | S | S | S | S | S | S | S | S | S | S | S | IV | No |
| 1. K945 | *S. epidermidis* | **R** | **R** | S | S | S | S | S | S | S | S | S | S | S | S | S | NT | No |
| 1. K292 | *S. epidermidis* | **R** | **R** | **R** | S | **R** | S | S | S | S | S | S | S | S | S | S | NT | **Yes** |
| 1. K862 | *S. epidermidis* | **R** | **R** | S | S | **R** | S | S | S | S | S | S | S | S | S | S | NT | No |
| 1. K619 | *S. epidermidis* | **R** | **R** | **R** | **R** | S | S | S | S | S | S | S | S | S | S | S | NT | **Yes** |
| 1. K536 | *S. epidermidis* | **R** | **R** | S | S | S | S | S | S | S | S | S | S | S | S | S | NT | No |
| 1. K445 | *S. epidermidis* | **R** | **R** | **R** | S | S | S | S | S | S | S | S | S | S | S | S | NT | No |
| 1. K339 | *S. epidermidis* | **R** | **R** | **R** | S | S | S | S | S | S | S | S | S | S | S | S | NT | No |
| 1. K314 | *S. epidermidis* | **R** | **R** | **R** | S | S | S | S | S | S | S | S | S | S | S | S | NT | No |
| 1. K350 | *S. epidermidis* | **R** | **R** | **R** | **R** | S | S | S | S | S | S | S | S | S | S | S | NT | **Yes** |
| 1. K1086 | *S. epidermidis* | **R** | **R** | S | **R** | S | S | S | S | S | S | S | S | S | S | S | NT | No |
| 1. K1081 | *S. epidermidis* | **R** | **R** | S | S | S | S | S | S | S | S | S | S | S | S | S | NT | No |
| 1. K297 | *S. epidermidis* | **R** | **R** | S | S | S | S | S | S | S | S | S | S | S | S | S | NT | No |
| 1. K256 | *S. epidermidis* | **R** | **R** | **R** | S | S | S | S | S | S | S | S | S | S | S | S | NT | No |
| 1. K218 | *S. epidermidis* | **R** | **R** | **R** | S | S | S | S | S | S | S | S | S | S | S | S | NT | No |
| 1. K196 | *S. epidermidis* | **R** | **R** | S | S | S | S | S | S | S | S | S | S | S | S | S | NT | No |
| 1. K163-3 | *S. epidermidis* | **R** | **R** | **R** | S | S | S | S | S | S | S | S | S | S | S | S | NT | No |
| 1. K1133 | *S. epidermidis* | **R** | **R** | **R** | S | S | S | S | S | S | S | S | S | S | S | S | NT | No |
| 1. K1129 | *S. epidermidis* | **R** | **R** | S | S | S | S | S | S | S | S | S | S | S | S | S | NT | No |
| 1. K1125 | *S. epidermidis* | **R** | **R** | S | **R** | S | S | S | S | S | S | S | S | S | S | S | NT | No |
| 1. K1071 | *S. epidermidis* | **R** | **R** | S | S | S | S | S | S | S | S | S | S | S | S | S | NT | No |
| 1. K980 | *S. haemolyticus* | **R** | **R** | **R** | **R** | **R** | S | S | S | S | S | S | S | S | S | S | I | **Yes** |
| 1. K940-1 | *S. haemolyticus* | **R** | **R** | **R** | **R** | **R** | S | S | **R** | S | S | S | S | S | S | S | I | **Yes** |
| 1. K901-3 | *S. haemolyticus* | **R** | **R** | **R** | **R** | **R** | S | **R** | S | S | S | S | S | S | S | S | I | **Yes** |
| 1. K900 | *S. haemolyticus* | **R** | **R** | **R** | **R** | **R** | S | S | **R** | S | S | S | S | S | S | S | I | **Yes** |
| 1. K418-2 | *S. haemolyticus* | **R** | **R** | **R** | **R** | **R** | S | **I** | **R** | S | S | S | S | S | S | S | I | **Yes** |
| 1. K14 | *S. haemolyticus* | **R** | **R** | **R** | **R** | S | S | S | S | S | S | S | S | S | S | S | II | **Yes** |
| 1. K63-2 | *S. haemolyticus* | **R** | **R** | **R** | **R** | **R** | S | **R** | **R** | S | S | S | S | S | S | S | III | **Yes** |
| 1. K217 | *S. haemolyticus* | **R** | **R** | S | **R** | **R** | S | S | S | S | **R** | S | S | S | S | S | III | **Yes** |
| 1. K752-1 | *S. haemolyticus /lugdunensis* | **R** | **R** | S | S | S | S | S | S | S | S | S | S | S | S | S | V | No |
| 1. K163-2 | *S. haemolyticus*  */lugdunensis* | **R** | **R** | **R** | S | S | S | S | S | S | S | S | S | S | S | S | NT | No |
| 1. K901-2 | *S. pasteuri* | **R** | **R** | S | **R** | **R** | S | **R** | **R** | **R** | S | S | S | S | S | S | I | **Yes** |
| 1. 3189 | *S. pasteuri* | **R** | **R** | **R** | **R** | **R** | S | **I** | **R** | S | **R** | S | S | S | S | S | I | **Yes** |
| 1. 3190 | *S. pasteuri* | **R** | **R** | **R** | **R** | **R** | S | **I** | **R** | S | **R** | S | S | S | S | S | I | **Yes** |
| 1. K963 | *S. kloosii* | **R** | **R** | **R** | **R** | **R** | S | S | **R** | S | S | S | S | S | S | S | I | **Yes** |
| 1. R21-2 | *S. kloosii* | **R** | **R** | **R** | **R** | **R** | S | **R** | S | S | **R** | S | S | S | S | S | III | **Yes** |
| 1. R33-2 | *S. xylosus* | **R** | **R** | **R** | **R** | **R** | S | **I** | **R** | S | **R** | S | S | S | S | S | I | **Yes** |
| 1. R23 | *S. xylosus* | **R** | **R** | **R** | **R** | **R** | S | **I** | **R** | S | **R** | S | S | S | S | S | IV | **Yes** |
| 1. K53 | *S. caprae* | **R** | **R** | **R** | **R** | **R** | S | S | S | S | S | S | S | S | S | S | II | **Yes** |
| 1. K501 | *S. lentus* | **R** | **R** | **R** | **R** | **I** | S | S | S | S | **R** | S | S | S | S | S | I | **Yes** |
| 1. K283-2 | *S. sciuri* | **R** | **R** | S | S | S | S | S | **R** | S | **I** | **R** | S | S | S | S | IV | **Yes** |
| 1. K627 | CoNS | **R** | **R** | S | **R** | **R** | S | **R** | S | S | S | S | S | S | S | S | I | **Yes** |
| 1. K284 | CoNS | **R** | **R** | **R** | S | **R** | S | **R** | S | S | S | S | S | S | S | S | I | **Yes** |
| 1. K901 | CoNS | **R** | **R** | **R** | **R** | **R** | S | **R** | S | S | S | S | S | S | S | S | II | **Yes** |
| 1. K528 | CoNS | **R** | **R** | **R** | S | **R** | S | S | **R** | S | S | S | S | S | S | S | II | **Yes** |
| 1. K919 | CoNS | **R** | **R** | **R** | **R** | **R** | S | **R** | S | S | S | S | S | S | S | S | III | **Yes** |
| 1. K213 | CoNS | **R** | **R** | S | **R** | S | S | **R** | S | S | S | S | S | S | S | S | III | **Yes** |
| 1. K938-1 | CoNS | **R** | **R** | S | S | S | S | S | **R** | S | S | S | S | S | S | S | IV | No |
| 1. K1055 | CoNS | **R** | **R** | S | **R** | S | S | S | S | S | S | S | S | S | S | S | IV | No |
| 1. K935 | CoNS | **R** | **R** | S | S | S | S | S | **R** | S | S | S | S | S | S | S | NT | No |
| 1. K651 | CoNS | **R** | **R** | **R** | **R** | **R** | S | S | S | S | S | S | S | S | S | S | NT | **Yes** |
| 1. K691 | CoNS | **R** | **R** | S | S | **R** | S | S | S | S | S | S | S | S | S | S | NT | No |
| 1. K475 | CoNS | **R** | **R** | **R** | S | S | S | S | S | S | S | S | S | S | S | S | NT | No |
| 1. K444 | CoNS | **R** | **R** | **R** | S | S | S | S | S | S | S | S | S | S | S | S | NT | No |
| 1. K301 | CoNS | **R** | **R** | S | **R** | S | S | S | S | S | S | S | S | S | S | S | NT | No |
| 1. K227 | CoNS | **R** | **R** | S | **R** | S | S | S | S | S | S | S | S | S | S | S | NT | No |
| 1. K173 | CoNS | **R** | **R** | S | **R** | S | S | S | S | S | S | S | S | S | S | S | NT | No |
| **Total R (%)** |  | **122 (100)** | **122 (100)** | **65 (53)** | **68 (55)** | **50 (42)** | **02 (2)** | **31 (25)** | **39 (32)** | **01 (0.8)** | **08 (6.6)** | **01 (0.8)** | **0** | **0** | **0** | **0** |  | **72 (59)** |

- R, resistant; S, susceptible; I, intermediate; MDR, multidrug resistance (isolate resistant to three or more classes of antimicrobials); NT, not type-able
- CoNS denotes other coagulase negative staphylococci whose species could not be determined in this study
- FOX, cefoxitin; PEN, penicillin; TET, tetracycline; SXT, trimethoprim-sulfamethoxazole; ERY, erythromycin; CHL, chloramphenicol; GEN, gentamicin; CIP, ciprofloxacin; RIF, rifampicin; CLI, clindamycin; MUP, mupirocin High level; TEI, teicoplanin; LIN, linezolid; VAN, vancomycin; DAP, daptomycin
- SCC*mec*, staphylococcal cassette chromosome *mec*, ‘a mobile genetic element of Staphylococcus species’.
