# Supplementary material for: Species and drug susceptibility profiles of staphylococci isolated from healthy children in Eastern Uganda
Source: PLoS One. 2020 Feb 13;15(2):e0229026. doi: 10.1371/journal.pone.0229026 (PMC7018018; doi:10.1371/journal.pone.0229026)
Supplement: Attachment — Submitted filename: Response to Reviewers.docx [file pone.0229026.s003.docx]

November 26, 2019

The Academic Editor, PLOS ONE

Dear Dr. Baochuan Lin, Ph.D.

RE: Submission of revised “PONE-D-19-21368”: Species and drug susceptibility profiles of staphylococci isolated from healthy children in Eastern Uganda.

We thank you for handling our manuscript through the peer-review process, and finding it suitable for publication in PLOS ONE.

We carefully considered and fully effected all the insightful comments from the peer-reviewers, as well as your specific concerns. We removed the awkward sentences in the manuscript. To the best of our ability, we have copy-edited the manuscript for language usage, spelling, and grammar. We hope that you will find our manuscript suitable for publication in your esteemed journal.

Again, we thank you and look forward to hearing from you soon concerning an editorial decision.

Yours sincerely,

David Patrick Kateete Ph.D.

Makerere University College of Health Sciences

Kampala, Uganda

POINT-BY-POINT RESPONSE TO THE REVIEWERS’ COMMENTS

SPECIFIC EDITORIAL COMMENTS:

1. The rational in abstract should be strengthened.

Response:

The rationale in abstract has been strengthened, see lines 19 to 28

2. Line 44 and 47, please use Staphylococcus aureus on line 44 and use S. aureus on line 47.

Response:

As advised, we have used Staphylococcus aureus and used S. aureus thereafter, lines 21 and 23.

3. Line 56, please spell out SCCmec since this is the first time it was mentioned.

Response:

As advised, we have spelled out SCCmec at first time of use, see line 76/77.

Journal Requirements:

1. When submitting your revision, we need you to address these additional requirements. Please ensure that your manuscript meets PLOS ONE's style requirements, including those for file naming. The PLOS ONE style templates can be found at http://www.journals.plos.org/plosone/s/file?id=wjVg/PLOSOne_formatting_sample_main_body.pdf and http://www.journals.plos.org/plosone/s/file?id=ba62/PLOSOne_formatting_sample_title_authors_affiliations.pdf

2. Please provide additional details regarding participant consent. In the ethics statement in the Methods and online submission information, please ensure that you have specified (1) whether consent was suitably informed and (2) what type you obtained (for instance, written or verbal). Since your study included minors under age 18, state whether you obtained consent from parents or guardians. If the need for consent was waived by the ethics committee, please include this information.

Response:

We affirm that our manuscript meets PLOS ONE's style requirements stipulated above, including those for file naming. We have also provided additional details regarding participant consent, see lines 96 to 104.

REVIEWERS' COMMENTS

REVIEWER #1:

Comment(s):

In this work, Kateete and colleagues determined the species and the antibiotic susceptibility of Staphylococci isolated from 764 healthy children under 5 years of age residing at the Iganga/Mayuge Health & Demographic Surveillance Site (IMHDSS) in Eastern Uganda. They isolated 513 staphylococci strains belonging to 13 different species. The most prevalent was Staphyloccoccus aureus, followed by S. epidermidis (25,5%), S. heamolyticus (2,3%), S. hominis (0,8%), and S. haemolyticus/lugduniensis (0.58%). The staphylococcal carriage rates from healthy children in rural Eastern Uganda were comparable to those from other countries showing a high level of virulence and AMR genes particularly in S. aureus strains.

Although restricted to a specific area in Africa, the identification of virulent staphylococci may offer some relevant information to the local clinical community, especially with the widespread occurrence of multidrug-resistant organisms and the increasing number of immunocompromised persons in the population. Continuous epidemiological surveillance would also contribute to reducing the Staphylococcal infection burden, hospital stay, and infection morbidity.

The manuscript is a well written, organized, and thorough presentation of the topic. I enjoyed reading the article and found it both relevant in its possible implications for the prevention and the development of targeted treatments and preventive strategies against invasive strains in children. There are some minor typos throughout the text, which can be easily corrected.

Response:

Thank you for the good remarks on our manuscript. We are delighted to hear that you enjoyed reading the manuscript, and that you found it informative regarding the widespread occurrence of antimicrobial resistance in context of immunocompromised individuals.

As advised, we have reworked the manuscript and eliminated the typos. The revised manuscript reads much better now.

REVIEWER #2:

Comment:

This study reports the prevalence of staphylococcal nasopharyngeal colonisation amongst healthy children in Eastern Uganda. 764 children were included, and the authors identified the species of each isolate, their antimicrobial susceptibility and also tested for some virulence and resistance genes.

The sampling appears to have been at a single time point, so “colonisation” should be used rather than “carriage” – which suggests sampling longitudinally. Secondly, only the nasal pharynx was sampled – so “colonisation” should be “nasopharyngeal colonisation” throughout.

Response:

We thank you for this and all your invaluable comments. As advised, we have now used the term “nasopharyngeal colonisation” throughout the text.

Comment:

Acronyms should be defined when they are first used. MDR defined line 170 – but used prior. A definition of MDR needs to be provided in the methods.

Response:

All acronyms are now defined at first use. A definition of multidrug resistance (MDR) has been provided in the methods (lines 130 to 131). We thank you.

Comment:

Proportions have been provided to 1, 2, and 0 decimal places. This needs to be consistent, and I would recommend using whole numbers only. P values should be reported as <0.05 (and or <0.001). There is no section to explain what tests were used to derive P values – nor what hypotheses were actually being tested. In some instances I don’t think the hypothesis testing is useful – e.g. it would be more useful to the reader to list the % of each species resistant to each antibiotic than provide the low P values, which is less informative. The statistical analysis needs to be described in the methods.

Response:

As advised, in the revised manuscript proportions have been provided consistently. Again, as you advised, we have used whole numbers only, and p-values are reported as suggested. Statistical analysis has been provided in the Methods section (lines 157-160), and it includes the specific tests we used to derive the p-values. We have used hypothesis testing only where we thought it would be useful. In Table 4 (pages 15/16) , we listed the percentages (%) in brackets for each species or isolates that was/were resistant or carrying a gene to each antibiotic. We thank you.

Comment:

How did the detection of resistance genes relate to the AST?

The genes listed should be consistent – LukSF or PVL is used (LukSF is more correct).

Response:

The genes investigated are now listed are now consistently used.

As you advised, we have used LukSF instead of PVL.

Regarding detection of resistance genes, apart from the mecA gene that correlated well with phenotypic methicillin/penicillin resistance, the detection of antibiotic resistance genes generally correlated poorly with antibiotic resistance. For example, of the 62 isolates that harbored the genes encoding the aminoglycoside-modifying enzymes (AMEs) (i.e. any of the aac(6')-Ie-aph(2'')-Ia, ant(4')-Ia and aph(3')-IIIa genes), only five (8%) were phenotypically resistant to gentamicin (an aminoglycoside). Additionally, although the vanA gene was detected in 20 isolates, phenotypic vancomycin resistance was not detected. Please note that such findings/observations have been reported before i.e. the detection of resistance genes may not always correlate with phenotypic resistance. We have added these additional aspects to the Results section and discussed the potential their implications in the Discussion section (see lines 241-247; 346-373). We thank you.

Comment:

From line 213 the isolates are compared to another set of isolates. There is no introduction as to where these isolates came from, apart from saying that they were from a hospital. Unless a lot of further information is provided about the provenance of these isolates and why comparison with the study isolates is important or interesting then this section should be removed.

Response:

Many thanks for raising this concern.

The reason why we compared isolates from the IMHDSS (current study) with previously characterized isolates from the hospital was an attempt to identify key differences in the distribution of virulence and antibiotic resistance genes, which could be used to distinguish between invasive/virulent and non-invasive staphylococci. We have clarified this, and we reworked the manuscript to this effect by providing additional information (see lines 82-93; 151-157; and 375-414). The is now an introduction as to where the isolates came from (lines 264-267 and earlier in the methods, page 7). This analysis was insightful as it showed that in Uganda, virulence and resistance genes are more prevent in hospital-associated isolates especially S. aureus at Mulago hospital compared to isolates from the community.

Comment:

In line 126 the genus name does not require italics.

Response:

The genus name has been un-italicized here, and throughout the manuscript. Thank you.

Comment:

Line 143 “MRS staphylococcus” is not correct

Response:

We agree. The phrase “MRS staphylococcus” is not correct and it has been revised, see lines 182-184. Thank you.

Comment:

MRS not defined until line 157.

Response:

This was an oversight. MRS is now defined in the Methods section where it is first used, see line 135.

Comment:

Line 276/277 virulence genes and resistance genes confused

Response:

Many thanks for pointing out this error. We have now separated virulence from resistance genes, please see lines 375 to 386.

Comment:

Line 280 does not with the preceding sentence.

Response:

This section has been clarified and it is now concordant, see lines 377-380. Thank you
