# Supplementary material for: Species and drug susceptibility profiles of staphylococci isolated from healthy children in Eastern Uganda
Source: PLoS One. 2020 Feb 13;15(2):e0229026. doi: 10.1371/journal.pone.0229026 (PMC7018018; doi:10.1371/journal.pone.0229026)
Supplement: Attachment — Submitted filename: Response to Reviewers.docx [file pone.0229026.s004.docx]

**January 12, 2020**

The Academic Editor, PLOS ONE

Dear Dr. Baochuan Lin, Ph.D.

**RE: Submission of revised PONE-D-19-21368R1: Species and drug susceptibility profiles of staphylococci isolated from healthy children in Eastern Uganda**

We thank you again for handling our manuscript through the peer-review process, and finding it suitable for publication in PLOS ONE.

We carefully considered and effected all the insightful comments from the peer-reviewers, especially Reviewer #2. We hope that you will find our revised manuscript suitable for publication in your esteemed journal.

For a detailed point-by-point response to reviewers, please see over.

Looking forward to hearing from you soon concerning an editorial decision.

Yours sincerely,

**David Patrick Kateete Ph.D.**

Makerere University College of Health Sciences

Kampala, Uganda

**POINT-BY-POINT RESPONSE TO THE REVIEWERS’ COMMENTS**

SPECIFIC EDITORIAL COMMENT:

Both reviewers agreed that the revised manuscript show significant improvement, however, there are still some issues/concerns that need to be addressed. Please review and address all comments from reviewer #2.

RESPONSE:

We have fully effected all the comments from Reviewer #2.

REVIEW COMMENTS TO THE AUTHOR:

**COMMENT:**

Reviewer #1: The authors have addressed all my suggestions. I found their responses quite satisfactory and the revised version has been much improved. I now recommend the paper for publication with no revisions.

**RESPONSE:
We are delighted to hear so. Thank you for taking time to review the manuscript.**

**COMMENT:**
Reviewer #2: The addition of some extra information about the hospital strains was helpful – but it is still inadequate. The methods needs to include information of how these were obtained, who from, what diseases they caused etc. If they’re from adults then careful explanation needs to be made to explain why a study of colonising strains from children would be compared to strains from a hospital obtained from adults. The two strain collections don’t really add up – all staphs vs. S. aureus and S. epi only. (My personal opinion is that this comparison actually doesn’t add a great deal to your manuscript).

**RESPONSE:
In the current revision, we have heeded your advice and removed the comparison between previously characterized hospital isolates and the current study. We thank you for the direction.**

**COMMENT:**

Line 57 … S. aureus strains are overwhelmingly coagulase-positive, some are atypical in that they do not produce coagulase.
This requires a reference to be added – ideally several references.

**RESPONSE:**

**References (See 5-11) have been added to this effect. Thank you.**

**COMMENT:**

Line 91 … “with the assumption that hospital-associated isolates are more invasive/virulent and/or resistant to antibiotics compared to community-associated isolates.” Why did you have this assumption? – prior research has shown that most hospital acquired S. aureus infections are endogenous – caused by the carriage strain. This might best be stated as the null hypothesis.

**RESPONSE:**

**In this revision, we heeded your advice and removed the comparison between previously characterized hospital isolates and the isolates in the current study as it was confusing. Thank you.**

**COMMENT:**

Table 4 still contains P values with a lot of decimal points, as does the text. Use 1 d.p. or use <0.05; <0.001. “S. haemolyticus was significantly more resistant to cefoxitin (p=0.0055), tetracycline (p=0.0041), SXT (p<0.0001), erythromycin (p<0.0001)…” should become: “S. haemolyticus was significantly more resistant to cefoxitin (p<0.05), tetracycline (p<0.05), SXT (p<0.001), erythromycin (p<0.001)”. These P values are low – but they don’t help the reader understand the message. It would be better to provide the proportions. These are NOT provided in table 1. So the text would read: “Resistance among S. aureus isolates to cefoxitin (x/y, z%), tetracycline (x/y, z%)…..was less common than S. haem isolates (cefoxitin a/b, c% resistant; tetracycline….). This type of information would be better in a table. Again – I don’t think the chi-sq testing is useful.

**RESPONSE:**

**We have revised the text as advised. We have provided proportions, and removed the p-values from the results as they are inconsequential. This information is summarized in Table 3, which we have cited in the results section. We hope that the text is now easier to understand. We thank you.**
